# Strategies for Identifying RNA Splicing Regulatory Motifs and Predicting Alternative Splicing Events

**DOI:** 10.1371/journal.pcbi.0040021

**Published:** 2008-01-25

**Authors:** Dirk Holste, Uwe Ohler

**Affiliations:** Whitehead Institute, United States of America

## Gene Expression and RNA Splicing

The regulation of gene expression is a ubiquitous phenomenon and is involved in virtually every process central to an organism, ranging from the fertilization of germ cells, across the cell cycle, to stimuli–response pathways or apoptosis. To control the expression of genes under such diverse contexts, regulation occurs on different cellular levels and involves a series of complex biochemical mechanisms that one can broadly classify into transcription, RNA processing and cytoplasmic transport, and post-transcriptional control and translation. While a series of distinct machineries is involved in controlling gene expression at each level, these complex circuits bear signs of interconnectedness [1].

**Figure 1:**
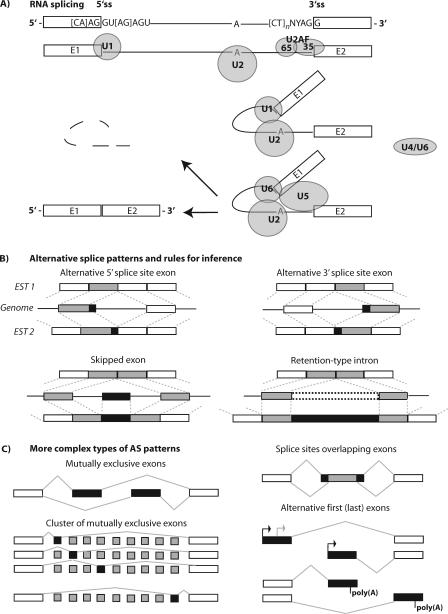
Basic Steps of Pre-mRNA Splicing and Patterns of Alternative Exons (A) Five small nuclear ribonucleoproteins (snRNPs U1, U2, U4, U5, and U6), an auxiliary splicing factor (U2AF), and many other factors (not represented) organized in the human spliceosome execute the excision of introns. After the recognition of 5′ss, 3′ss, and branch point, respectively, by U1, U2AF, and U2, the intron is first cleaved at the 5′ss and subsequently at the 3′ss, mediated by U4/U6 and U5 (U1, U2, and U4 are detached later during the cycle). The intron remains in the nucleus and is degraded, while ligated exons are transported outside to the cytoplasm. (B) AS events can be inferred by spliced alignments of mRNAs to genomic DNA (cf. Figure 3), indicated by dashed lines (AS part of exon colored in black), and commonly distinguished in terms of whether mRNA isoforms differ by skipping of an exon (SE), or whether isoforms differ in the usage of a 5′ss or 3′ss, producing an A5E or A3E, respectively. A fourth type, termed retention-type intron, occurs when two isoforms differ by the presence of an unspliced intron in one transcript that is spliced in the other. (C) More complex types of AS forms can be constructed from canonical splice variants; different isoforms can also be the result of variations at the 5′- and 3′-terminus of transcripts, which are not necessarily due to AS.

In higher eukaryotes, splicing constitutes a critical mode for the regulation of gene expression at the level of RNA processing [2–4]. The large majority of eukaryotic protein-coding genes are transcribed as precursors of messenger RNAs (pre-mRNAs), in which exons are separated from each other by intervening regions of non-protein–coding information (introns), which have to be correctly spliced out to produce a mature mRNA. Splicing of pre-mRNAs occurs in a two-step reaction (Figure 1). In the first step, the message is cleaved at the 5′ end of an intron, and this 5′ end is linked to the branch point, which is typically in close proximity upstream of the 3′ end of the intron. In the second step, the mRNA intermediate is cleaved at the 3′ splice site (3′ss), exons are ligated, and the intron lariat is released [2]. During later stages of spliceosome assembly, the 5′ss and 3′ss pair and interact (typically across the exon, but pairing across an intron can occur), supported by general and specific splicing factors that recognize them. Typical mammalian genes span tens of thousands of nucleotides, with on average nine exons and protein-coding regions on the order of a thousand nucleotides, thus embedding “exon islands” within a large “sea” of noncoding nucleotides that have to be accurately recognized for correct splicing and exon ligation. This important task is executed in the nucleus by the spliceosome, a large ribonucleoprotein (RNP) complex that involves five small nuclear RNAs and potentially hundreds of proteins, the core components of which are highly conserved across metazoan genomes [5].

**Figure 3:**
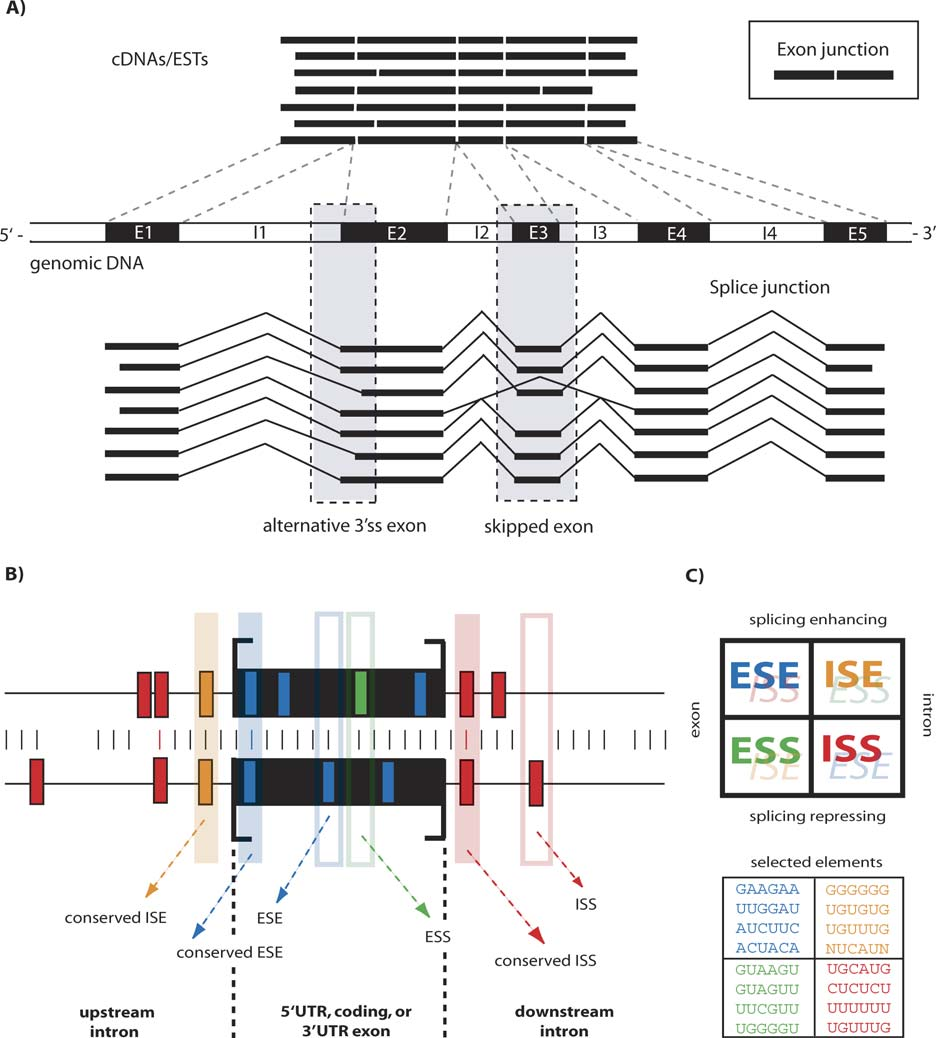
From Sequences to Patterns and Functional Elements (A) AS events can be computationally inferred by spliced-sequence alignments of complete or partial mRNAs to genomic DNA. A selection of available algorithms and software is listed in Table S1. The sketch shows seven mRNAs with indicated exon junctions (for visual guidance only), the primary transcript structures of which are to be inferred from alignments to genomic DNA (the order of the mRNAs above and below the genomic DNA is the same). In the example shown, the set of mRNAs aligns to five exons (E1 to E5), and the data are consistent with two AS events: E2 alternative 3′ss splicing, and E3 skipping (skipped in the fourth mRNA from the top). (B) Splicing-regulatory elements are distinguished depending on their location (exon or intron) and their mode of action (enhancing or silencing): 1) exonic splicing enhancer (ESE) elements; 2) exonic splicing silencer (ESS) elements; 3) intronic splicing enhancer (ISE) elements; and 4) intronic splicing silencer (ISS) elements. One can subclassify these elements whether they carry protein-coding information, act in the context of 5′ss and/or 3′ss, or are sequence-conserved across species (indicated by the presence of vertical colored bars). (C) Often, ESE, ESS, ISE, and ISS elements do not act independently of their sequence context, but can assume antagonistic functions (enhancing versus silencing) in splicing. The color-coded example sequence elements are taken from the literature [27,28,32,38].

Signals that specify exon–intron junctions are located at the termini of introns. *cis*-Acting nucleic-acids elements are located at the 5′ss, branch point, and 3′ss, and guide the spliceosome. Almost all introns are characterized by /GT and AG/ termini at the 5*'*ss and 3*'*ss, respectively (U2-type introns). In addition to the canonical /GT and AG/ termini, between four and seven nucleotides (5′ss) and up to about 20 nucleotides (3′ss) typically contain information for splicing. A small fraction of U2-introns exhibits /GC–AG/ termini, while a tiny fraction exhibits /AC–AT/ termini (U12-type introns). U2-type and U12-type introns are spliced by distinct spliceosomes [2]. In addition to the precise recognition of exon–intron junctions among many possible pseudo-splice sites (that is, intronic nucleotides matching the splice site consensus) and the splicing of introns, the spliceosome also has to integrate this with other steps in RNA processing, such as capping, cleavage, and polyadenylation [6]. A picture emerges in which the control of gene expression is in part thought of as a network of interactions between transcription and RNA processing, export, and transcript quality control [1].

### 

#### One gene, different messages.

**Figure 2:**
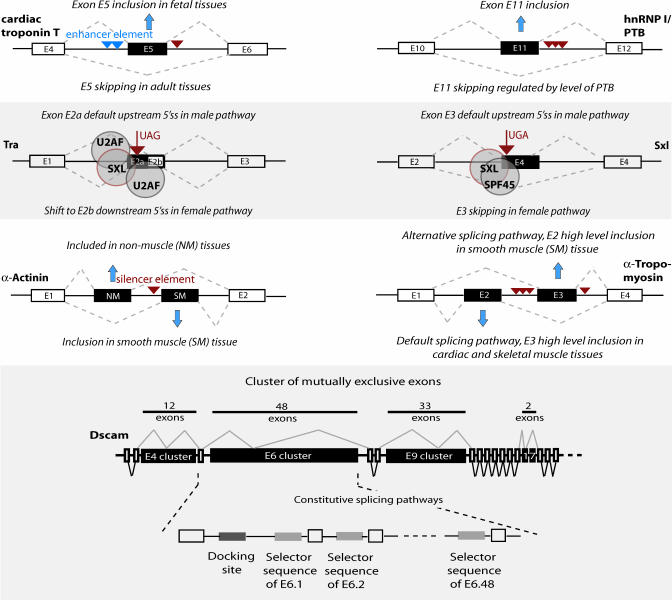
Selection of Splice Patterns of Known Alternative Exons Selection of splice patterns of known alternative exons of *Tra*, *Sxl*, and *Dscam* genes in D. melanogaster [3,4], and *α-Actinin*, *α-Tropomyosin*, *Troponin-T*, and *PTB* in H. sapiens [71]. Exon skipping is the predominant AS event in many metazoans and, e.g., has been shown to be involved in tissue- and developmental stage–specific regulation, as well as autoregulation (*PTB*) [72]. AS products of pre-mRNAs expressed from *Tra* and *Sxl* genes are involved in the pathway of somatic sex fate in D. melanogaster, which is regulated by altogether five AS genes at the top of the determination cascade [4]. The “master gene” *Sxl* is expressed in female flies, where it acts as a negative regulator of splicing. AS of the *Dscam* gene is known for its theoretically large number of possible different AS products (∼38,000 against ∼14,000 D. melanogaster protein-coding genes), which are derived from four clusters of skipped exons. The regulation of one cluster includes so-called selector-docking sites, which are inverse complementary overlapping sites located in the most 5′-end intron (docking) and upstream of each skipped exon (selector) of this cluster, respectively [73].

The splicing pattern of many pre-mRNAs is variable: different splice sites may be used as alternatives, giving rise to multiple alternatively spliced (AS) mRNA isoforms, and thus producing mature mRNAs and ultimately polypeptides that can be highly similar or markedly different while originating from the same locus [2–4]. Detailed molecular studies of regular and disease-associated genes have identified several hundred genes subject to AS. One powerful but not typical example of the possibilities opened up by the process of AS is given by the D. melanogaster gene *Dscam* (Figure 2), which has the potential to produce and express hundreds to thousands of alternative mRNA isoforms [7]. Computational analyses of available large datasets of spliced mRNAs to genomic DNA infer that a large number of mammalian genes (often estimated at more than 50%) produce messages that are consistent with variable splice site choices made during alternative pre-mRNA splicing [8,9]. In fact, the average number of detected isoforms per gene can reach such an extent that one can hardly distinguish the “alternative” transcript and might instead invoke the concept of a set of transcripts produced from a gene's locus [10]. Such whole-genome bioinformatics studies have added further lines of evidence to the occurrence and scope of AS, such that it is now considered to be critically contributing to the diversification of proteins expressed in different cell types and developmental stages. As splicing is critical to the viability of the cell, it is clear that nonphysiological splicing decisions can have pathological effects, and consequently splicing and AS are gaining interest as possible explanations for human genetic disorders [11]. Figure 2 displays splice variants of known AS genes, originating from exon-skipping, alternative 3′ss exons, or mutually exclusive exon splicing.

In addition to protein diversification, AS might have another function in the realm of gene regulation, by linking splicing to a downstream control mechanism, termed nonsense-mediated mRNA decay (NMD) [12]. Under this scheme, aberrant or deliberately produced mRNA isoforms that harbor shifts of the original reading-frame and hence lead with high probability to premature termination codons downstream of the frame shift, are candidate substrates for NMD and shutting down of protein synthesis. While computational studies have inferred a significant and large number of possible NMD target isoforms [13], first interrogations of splicing-sensitive micro-arrays and NMD mutants have so far failed to detect large support for a widespread utilization of this mechanism [14].

#### Computational challenges.

As experimental systems and models for the regulation of AS have been steadily validated and refined, so have bioinformatics tools and computational models [15,16]. With the availability of complete genome sequences and comparative genomics, the identification of candidate sequence elements that can be evaluated for their activity in controlling gene expression has become a major challenge in computational molecular biology. In order to systematically address the level of complex control achieved by AS, experimental and computational large-scale studies have started to illuminate the extent, structure, and regulatory consequences of AS and its differential usages in mammalian genomes. Here, we focus on two selected aspects out of a large body of computational approaches, which have been at the center of several recent studies: starting with basic steps for data acquisition and splice patterns classification, we first present an overview and compare several methods for candidate splicing *cis*-regulatory element detection. Subsequently, we move toward the goal of predictive identification of AS events from genomic sequence. There are many additional aspects of AS worth mentioning, ranging from specific algorithms for spliced alignments to the population genetics of exon and intron evolution, and for these we would like to refer to additional recent excellent reviews [4,17–20].

## From Transcripts to Patterns of Alternative Splicing

The principal computational approach to identifying AS genes, and to infer individual alternative exon events or complete alternative isoform structures, relies on the comparison of available transcript data to assembled genomes and known gene loci. For this framework to work well, one needs available complete and annotated genomes, large collections of transcribed sequences acquired under various cellular contexts (e.g., different cell or tissue types), and reliable and efficient algorithms for sequence alignment. In an initial step, large-scale alignments of transcripts to genomic DNA are conducted using a variety of systems (a selection is listed in Table S1). The genomic sequence is usually of high-end quality, whereas transcribed sequences come in two different flavors: 1) complementary DNAs (cDNAs), which often produce a single or at least very limited number of possible genomic matches; and 2) expressed sequence tags (ESTs) or shorter reads from massively parallel sequencing, which can produce a considerably larger number of possible matches. ESTs are sequenced in a single pass and are therefore available in large numbers, but often quite error-prone especially toward the ends of a read.

At the heart of spliced-alignment algorithms are often dynamic-programming approaches: given a transcribed sequence (the mRNA), provide an alignment of a second contiguous sequence (the genomic DNA) to it that is allowed to be interrupted by long gaps which correspond to spliced-out introns (Figure 3A). Standard gap opening/extension penalties are not appropriate in this context; rather, gap penalties should be based on intron length distributions, and gaps should preferentially appear at positions that correspond to splice sites. Practically, such an alignment is most feasible when the contiguous sequence is restricted to genic DNA (e.g., ensembl- or similarly known annotated genes). In the context of millions of available EST sequences, some systems also use shortcuts to avoid the often-prohibitive quadratic runtime complexity. The work flow directing the genoa system [10] can serve as a practical example and is similar to those in many databases: i) find candidate matches of identity between (repeat-masked filtered) cDNA sequences and genomic DNA; ii) determine spliced-alignments of significant matches of cDNAs to gene loci; iii) find matches of EST to successfully aligned cDNAs; and iv) splice-align significant matches of ESTs to previously cDNA-aligned gene loci. Afterward, quality filters are typically applied, e.g., on the number of hits of cDNAs/ESTs, percent cDNA/EST sequence aligned and sequence identity, minimum exon and intron sizes, maximum intron size, canonical splice sites, or the exclusion of genes that are subject to frequent DNA rearrangements (e.g., immunoglobulin genes).

After the genome-wide alignment, the annotation of constitutive and alternative exons is the next step. All transcripts aligned to a gene's locus are scanned for AS events, to identify alternative isoforms and which exons or introns they affect. In this framework, constitutive exons are the “default” status of exons, and this status remains unless specified conditions for annotation as an alternative exon are met. To this end, one can order observed splice junctions (SJs) and their frequency of occurrence, and construct an SJ matrix—with exons considered as “nodes” and SJs as “edges” connecting nodes. A traversal through this graph can capture different AS patterns [21]. This scheme is exon-centric, in that splice patterns are individually evaluated for each exon. Individual events are commonly categorized in four canonical patterns (Figure 1B), referred to as “skipped exon” (SE, or cassette exon), alternative 5*′*ss (A5Es) or alternative 3*′*ss exons (A3Es), or retention-type intron. These descriptions are not necessarily exclusive, and an exon can make several alternative splice site choices. Exon-centric schemes can only detect canonical events, but can be extended to capture more complex events, such as mutually exclusive exons or clusters of skipped exons (Figure 1C). In order to pair-wise compare isoforms generated from one gene against one another, a possible heuristic solution is to capture the total number of SJs that differ between two transcripts and normalize it to the total number of SJs, within a region where both transcripts overlap genomic DNA [22]. Storing ingoing and outgoing edges in the SJ matrix allows for the construction of complete isoforms as a representative path through the graph [16].

Computational analyses indicate that AS predominantly generates SE events in both human and mouse transcriptomes [22], and likely generally across vertebrates [23]. The frequency of SE events is followed by A5E and A3E events, which in turn are followed by retention-type introns, overlapping exons (simultaneous occurrence of A5E and A3E events), and mutually exclusive exons. When accumulating splice variants and monitoring the respective frequency of alternatives, one often obtains a bimodal distribution of the percentage of events, in particular for the inclusion and exclusion of SEs, and the two variants are in such cases referred to as “major” and “minor” isoforms depending on their frequency. Expectedly, the availability of a larger number of cDNAs and ESTs from a gene increases the chance of observing alternative isoforms of that gene, so the proportion of AS genes will tend to increase with increasing transcript coverage of genes. Probabilistic and sampling strategies have been discussed to circumvent or correct for this bias [21,22,24].

## Signals for Splicing Specificity and Strategies for Their Identification

A comparison of general splicing signals (5*′*ss, 3*′*ss, and branch point) between baker's yeast, C. elegans, A. thaliana, and humans shows that the information content of these signals is less and less preserved with an increasing number of introns [25,26], and in higher eukaryotes is often insufficient to ensure correct RNA splicing. The growing degeneracy, in turn, opens up the possibility for making alternative splice sites choices, and additional signals become necessary [27], in particular when weak splice sites are involved [28]. It is known that the choice of a splice site can be affected by a number of features, including exon and flanking intron size, splice site strength, splicing regulatory elements, interspersed repeat content, mRNA secondary structure, or RNA editing. Splicing-specific elements function in context as exonic/intronic splicing enhancers (ESE/ISE elements) or silencers (ESS/ISS elements), and alter splice site choices by recruiting positive or negative *trans*-acting regulatory factors (Figure 3B). All elements are more or less ubiquitous in constitutive and alternative exons, short (∼6–10 nucleotides long, with some longer exceptions), and present in the majority of exons or introns [29,30].

While there is presently no validated large set of such elements, tested in various standard splicing contexts, ESE and ESS elements are currently the best characterized [17,31]. ESE elements often recruit arginine/serine dipeptide-rich (SR) proteins, which themselves recruit other spliceosomal components via protein–protein interactions. The family of nuclear heterogeneous RNPs (hnRNPs) characterizes another class of splicing factors that often antagonizes members of the SR protein family and are thought to be recruited by ESS elements. Biochemical investigations have further revealed highly specific factors that bind to splicing regulatory elements, including members of the CELF [32], NOVA [33,34], PTB [35], and FOX [36] families of proteins. FOX-1, for example, is an RNA-binding protein expressed in brain, heart, and skeletal muscle tissues, and binds to the UGCAUG motif [37]. Interestingly, this motif has been computationally identified downstream of the 5*′*ss region, by searching AS genes specifically expressed in the human brain displaying exon-skipping events [38].

Early indications for the role of splicing regulatory elements were obtained, e.g., from disease-associated studies where a chance disruption pointed to the presence of functional sites, and often to evolutionary conservation around sites. Subsequently, several systematic computational and/or experimental assays have been developed to identify ESE, ESS, and other elements, and they can be grouped into the following main classes:

1. A **functional SELEX (**systematic evolution of ligands by exponential enrichment) approach was employed to search for ESE elements [39]. Using repeated rounds of selection, a library of random oligonucleotides was inserted into a minigene construct of known splicing behavior, replacing an authentic ESE. An entire pool of constructs, each with a different random oligonucleotide, was transcribed and spliced to produce a pool of mRNAs. For the next round, products of spliced mRNAs were amplified and used to construct a new generation of minigenes, thus starting another cycle. After several rounds, the “fittest” sequences with the desired splicing activity were sufficiently enriched and could then be extracted by sequencing. Subsequently, motif searches and/or multiple alignments are used to construct scoring matrices, e.g., for ASF/SF2, SC35, SRp40, or SRp55 [39,40].

2. A **splicing reporter system** was developed in [27] to systematically screen for ESS elements. To this end, a three-exon (E1-E3) minigene construct was designed, where first and last exons together encode the complete green-fluorescent protein (GFP) and the test exon (E2) is located between E1 and E3. Two states were of interest: 1) when E2 was included into the mature transcript, the resulting protein was not functional; 2) when E2 was skipped, E1 and E2 formed functional GFP. Using a library of random oligonucleotides, which were individually inserted into E2, test constructs were transfected into cultured cells. The cells were then automatically screened for signals of GFP-expression by fluorescent-activated cell sorting (FACS), and GFP-active cells could then be extracted for sequencing.

3.** Computational identifications of candidate splicing-regulatory elements** in exons and introns on a genome-wide scale have been conducted, and they can be grouped into searches for elements involving one species, or comparative searches in genomes of related species (Table S2). rescue-ese elements [28] were identified by statistical analyses of exons, flanking intron regions, and splice site composition. Building on the observation that ESEs can compensate for weaker 3′ss and/or 5′ss of constitutive exons, ∼240 human rescue-ese motifs were predicted in a large exon set, by selecting hexamers that were enriched in exons against introns and weak against strong splice site scores. To further validate rescue-ese–predicted motifs, a population genetics strategy (verify) was developed [41] to assess the extent of purifying selection on functional sequences. Using a large collection of human single-nucleotide polymorphisms (SNPs), verify estimated that about one-fifth of mutations disrupting rescue-ese elements were eliminated by selection.

pese/pess elements (putative exonic splicing enhancers/silencers) were similarly identified, but come with a different flavor by avoiding any potential bias resulting from codon usage [42]. Here, the frequency of occurrences of oligonucleotides in noncoding exons was contrasted against pseudo-exons and 5′-UTRs of intronless genes. Oligonucleotides that were sufficiently overrepresented in noncoding exons were selected as pese elements, while underrepresented ones were selected as pess elements.

esr (exonic splicing-regulatory) and isr (intronic splicing-regulatory) elements were identified in comparative analyses [43,44]. To this end, the former approach used a comparison of the frequency of expected against observed codon pairs (hexamers), which were additionally highly conserved in the codon wobble positions between exons of orthologous H. sapiens and M. musculus genes. The latter approach focused on four-way conserved oligonucleotides in 400 nucleotides long intronic regions upstream and downstream of all flanking exons, by including the additional mammalian genomes of C. familiaris and R. norvegicus. Statistically enriched oligonucleotides were retained and clustered into groups based on their conservation. A related approach to search for regulatory elements in two nematodes, C. elegans and C. briggsae, was used in [45].

Out of a total of 4,096 different hexamers, the above searches predicted elements that span a range between several hundred to more than 50% of all hexamers, some of which overlap to a large extent. This raises the question of how much information is already contained in these sets, and which splicing-regulatory elements possibly remain to be predicted. An approach to group-predicted motifs was developed in [46], based on compositional differences (“distances”) between elements. It inferred both ESE and ESS (ni-ese/ni-ess) elements from a compendium of rescue-ese, fas-ess, and pese/pess elements, by using the sequence similarity to known ESE and ESS hexamers and a discriminating function to group between positive, negative, and splicing-neutral activity. Table S3 shows an all-against-all sequence comparison between the different sets of predicted regulatory elements, and Dataset S1 collects all these splicing *cis*-regulatory elements discussed above.

Several lines of evidence suggest that the influence of *cis*-regulatory elements exerted on splice site choices is context-dependent, and consequently the label “ESE” (or any other) is correct only in so far as the context is considered. An ESE element may well act as negative regulator of splicing in another context, e.g., when inserted into flanking intron regions or other exons (cf. Figure 3C).

## Computational Models and Methods for the Prediction of Alternative Splice Site Choices from Sequence

### 

#### Phylogenetic conservation of alternative splicing.

The identification of AS events has traditionally been based on the analysis of the diversity observed in transcribed sequence data. As gene expression is largely condition-specific, it is hard to predict when we will have generated and sequenced EST libraries under sufficiently different conditions, and sufficiently deep to arrive at a complete compendium of transcript diversity. This brings up an additional caveat: noise occurs both on the level of experiment as well as in the cell. Filters imposed on EST-based AS inference help to reduce experimental noise and eliminate unwanted contamination. Yet, given the considerable degeneracy of sequence signals for splicing, the machinery itself is inherently noisy and prone to (reproducible) errors, and cellular mechanisms such as NMD have evolved to eliminate erroneously spliced products. As it has been provocatively put, one may be able to observe the alternative use of any possible splice site if one just sequences enough ESTs [47]. The question therefore is how many AS events actually correspond to a specific function. In any case, widespread AS may serve as “evolutionary tunneling” [9,48] and provide an organism with a mechanism to quickly “explore” new isoforms, only few of which eventually become functional and get fixed. In a larger context, this connects to the question of the evolution of AS and the structure of eukaryotic genes per se [19,49].

**Figure 4:**
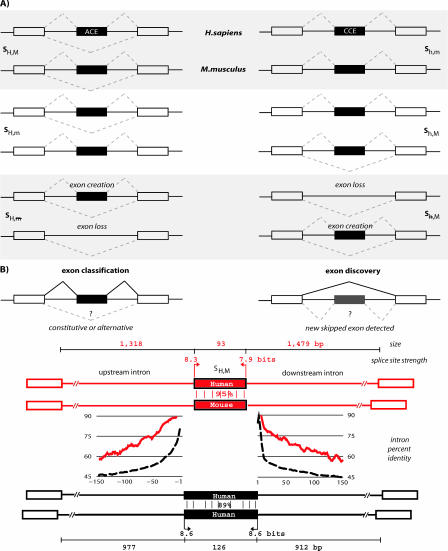
Conservation of AS across Species (A) Splice patterns of exons of pairs of orthologous genes can be classified into four “pattern conservation” categories, demonstrated here for SE events in *H. sapiens/M. musculus*: both exons are constitutively spliced (S_h,m_)—known as “constitutive conserved exons” (CCEs); the exon of the human gene is alternatively spliced, the mouse one constitutively (S_H,m_); the exon of the mouse gene is alternatively spliced, the human one constitutively (S_h,M_); both exons are alternatively spliced (S_H,M_)—known as “alternative conserved exons” (ACEs). In addition, one can define two “gain/loss” categories as: the exon of the human gene is alternatively spliced, the mouse exon is absent (S_H, m_); the exon of the mouse gene is alternatively spliced, the human exon is absent (S_h,M_). (B) AS events can be successfully predicted ab initio, that is, from genomic sequence alone. The figure refers to SEs in particular, but results for other classes of AS events have also been described. Published approaches address one or both of two problems: exon classification and exon discovery. Constitutive and alternative exons show different characteristics, which can be used as features for nontranscript methods. Example features include length of the AS exon and surrounding intron; strength of the splice sites; the level of conservation in the exon and surrounding introns; and coding-typic conservation patterns in exon sequences. In addition, some approaches utilize the occurrence of specific sequence features corresponding to splicing regulatory elements (parts of the figure adapted from [50]).

To reduce spurious nonfunctional alternatives, conservation was initially used as a filter. More recently the focus of comparative genomics has shifted toward identifying AS events that are 1) conserved with respect to orthologous genes (“alternative conserved exons”, or ACEs); 2) only alternatively spliced in one species; or 3) newly created alternative exons, which are absent in orthologous genes of other lineages (Figure 4A). Given the differences in primary datasets and protocols for the inference of AS, it is difficult to estimate the fraction of splicing-conserved exons of orthologous genes. Based on transcript-inferred and predicted ACEs, the proportion of ACEs shared between H. sapiens */* M. musculus is estimated to be ∼11% of all EST-derived skipped human exons [50], while other estimates determine about 50% or even higher levels of conservation [51,52]. Possible reasons for such differences point to a lack of standards across different datasets and databases, and could possibly be attributed to differences in cDNAs/ESTs, inference of constitutive and/or alternative exons, mixing of splice variants (in terms of alternative splice site usage, or high- and low-frequency inclusion), orthologous gene relationships, or stringency of comparative analyses.

#### Ab initio prediction of alternative splicing events.

In response to incompleteness and noise issues that come with transcript-derived isoforms, recent years have seen a number of approaches that aim at the direct “ab initio*”* identification of AS isoforms, by methods that solely rely on (comparative) sequence information of genomic DNA alone, without additional data such as expressed sequences or protein information. This is possible because exons affected by AS have different characteristics when compared to constitutive ones. In mammals, exon-skipping is the predominant type of AS, and sets of ACEs of orthologous human–mouse genes could be identified and analyzed for such functional characteristics [53]. Compared to constitutive conserved exons, ACEs are on average shorter, have weaker splice sites, and exhibit higher sequence-conservation of the exon body and flanking intron regions [53], lower ESE frequencies [40] (Holste, unpublished data), fewer SNPs, and are under higher natural selection pressure [47]. They are also more likely to preserve the reading frame and less likely to disrupt protein domains, are enriched in genes expressed in the brain, and in genes involved in transcriptional regulation, RNA processing, and development [50]. Several “non-transcript-based” algorithms have utilized these observations to identify new AS isoforms, and they can be grouped into two main classes addressing related but different problems:

1. **Exon *classification* algorithms** take known exons as input and classify them into “constitutive” or “alternative”. Typically, a classifier is built on known examples of constitutive and AS exons, and uses sequence features extracted from the exon and surrounding introns. The approaches frequently work on known pairs of orthologous exons*,* as conservation features strongly contribute to the performance of the classifier. An exception is the single-species approach of [54], which was applied to the genome of C. elegans, and where the missing information from conservation is arguably offset by the simpler genome organization compared to mammals.

One of the first such algorithms used only exon and flanking intron conservation as features to predict that a given conserved exon was subject to any form of AS in two species of *Drosophila* [55]; despite the simplicity of the features, its specificity was more than 40%. At the same time, the first system to detect ACEs in the mammalian genomes combined thresholds of exon and intron conservation with exon size and frame conservation, and achieved a sensitivity of ∼32% with virtually no false positives [56]. The sensitivity of these simple approaches can be dramatically improved by using a larger set of features, e.g., the presence of oligonucleotides corresponding to known or predicted sequence elements, combined with solid machine learning approaches that include the selection of significant features from the large set of motifs or the utilization of sparseness priors [50,54,57,58]. The acescan system, for instance, reported an equal recognition rate (balanced sensitivity and specificity) of ∼90%, while the specificity of newly predicted ACEs was ∼70% in experimental validations [50].

2. **Exon *discovery* algorithms** take introns as input and parse them for the presence of hitherto unknown (and thus presumably mostly skipped) exons. This class has received somewhat less attention; the methods are generally based on gene finding–related approaches such as pair hidden Markov models (e.g., in the uncover system [59]), which parse a pair of orthologous input sequences into segments with different functions and/or patterns of conservation, such as splice sites or coding triplets [60]. This approach has been extended to utilize multiple alignments of several species of *Drosophila* [61].

The exons in classification algorithms are known already, and this usually implies that the major isoform is exon inclusion; in comparison, newly discovered exons tend to be excluded in the majority of transcripts, which explains why they have not yet been annotated. Figure 4B exemplifies the differences of the two approaches, as well as some of the typical features used by the classifiers.

Current algorithms mostly deal with the case of SEs; however, other AS types have been tackled as well. In addition to identifying new ACEs, the uncover model also allows us to predict cases of conserved coding H. sapiens */* M. musculus retention-type introns [59]. An early approach focused on the identification of new partners in a mutually exclusive pair of SEs [62]; these exons often arise from local duplications and therefore share considerable sequence similarity. Additional models use protein domain information to identify which newly predicted AS isoforms, generated by exon skipping and/or retention-type introns, would generate known protein domains [63,64].

How much do these exons contribute to the overall variability in gene structures? acescan (an exon classification method) predicted ∼4,000 exons to be ACEs in the human genome [50]; uncover (an exon discovery algorithm) predicted ∼50 new splicing-conserved SEs in human ENCODE regions [59] and ∼8,500 exons genome-wide, which are annotated neither in H. sapiens nor in M. musculus. When coupled with acescan, more than 6,000 of these passed as putative AS candidates (Ohler and Yeo, unpublished data). A similar survey used the phyloHMM exoniphy to arrive at ∼700 new AS exons—an order of magnitude less, possibly due to initial conservation requirements in several additional mammalian species [65]. Concerning retention-type introns in coding regions, uncover predicted the surprisingly low number of two-dozen conserved retention-type introns across H. sapiens–M. musculus orthologous protein-coding genes. In accord with this small number, Hiller et al. [63] identified 65 coding retention-type introns, but with the majority involving noncanonical splice sites not modeled in uncover. A direct comparison of transcript-derived retention-type events was also indicative of low conservation (Nostrand, Holste, and Burge, unpublished data). Overall, Sorek et al. estimated that ∼7% of coding exons undergo some type of AS conserved between H. sapiens and M. musculus [65]. Even if each of these variants would affect a different gene, this identifies a considerably sized gap between EST-observed and species-specific AS events on the one side, and conserved and presumably functional AS events on the other side.

None of these algorithms predicts complete mRNA isoforms; rather, they provide the building blocks of these by identifying the parts of a gene susceptible to different mechanisms of AS. Rare instances of ab initio gene-finding algorithms allow for predicting several complete alternative gene structures in the input sequence [66,67]; these algorithms are able to enumerate possible gene structures, but do not use information of functional splicing *cis*-elements or *trans*-factor concentrations to arrive at AS isoforms which are actually produced. A “splicing simulator” would take as input the sequence of a pre-mRNA and be able to automatically predict which isoforms exist and, with additional context information such as the expression levels of all splicing factors, how frequently they are generated. Compared to computational gene finders, which strongly rely on statistical properties of reading frame, coding content, and phylogenetic conservation [67], such an approach would only make use of the information the cell has available at the time of splicing in the nucleus. In practice, such splicing simulators are still in early stages of development, but preliminary successful results have already been achieved with approaches that combine models for splice sites explicitly [27] or implicitly [68] with other splicing regulatory motifs such as ESE and ESS elements.

## Resources

Finally, we want to point to resources available to researchers who wish to obtain a deeper understanding of the transcript variability within genes of interest, and how this variability is generated and regulated on the level of RNA splicing.

### 

#### Value-added databases.

Databases for recording types of AS have been designed and operated for some time now, and they can be grouped into two approaches: 1) based on searches of published research [69,70]; and 2) automated large-scale comparisons of transcript sequences. Broadly speaking, the first approach emphasizes the manual curation and focuses on the “specificity” of (authentic) AS events, while computational approaches have their focus on “sensitivity” as well. Currently, the pipelines to annotate gene structures in these databases (and even more so in general-purpose genome browsers) are heavily driven by EST and homology evidence, and do not provide information on splicing regulatory elements or ab initio–predicted AS events or mRNA isoforms. AS databases that begin to include such information are becoming available and provide a more comprehensive picture of splicing and its regulatory elements (Table S4).

#### Bioinformatics software and Web servers.

Several of the systems discussed here are accessible through a Web server or available as download for local analyses (cf. Table S5 for a selected overview).

## Supporting Information

Table S1 Algorithms for Spliced-Sequence Alignments To Infer Primary Transcript Structures (29 KB PDF)

Table S2 Overview of Comprehensive Motif Searches for Splicing-Regulatory Sequence Elements (42 KB PDF)

Table S3 Comparison of the Different Classes of Predicted Regulatory Elements Described in Table S2
(45 KB PDF)

Table S4 Databases for Alternative Splicing (59 KB PDF)

Table S5 Algorithms for Ab Initio Detection of Alternative Splicing Events (32 KB PDF)

Dataset S1 Collection of All *cis*-Regulatory Elements with Predicted Splicing Enhancing or Silencing Activity Reviewed in This Article (1.4 MB XLS)

## Abbreviations

3′ss: 3′ splice site
5′ss: 5′ splice site
A5E: alternative 5′ss exon
A3E: alternative 3′ss exon
AS: alternative splicing or alternatively spliced
cDNA: complementary DNA
ESE (ESS): exonic splicing enhancer
EST: expressed sequence tag
NMD: nonsense-mediated mRNA decay
SE: skipped exon
SJ: splice junction.
