# Supplementary material for: Strategies for Identifying RNA Splicing Regulatory Motifs and Predicting Alternative Splicing Events
Source: PLoS Comput Biol. 2008 Jan 25;4(1):e21. doi: 10.1371/journal.pcbi.0040021 (PMC2217580; doi:10.1371/journal.pcbi.0040021)
Supplement: Table S1 — (29 KB PDF) [file pcbi.0040021.st001.pdf]

Table 1. Algorithms and software for spliced-sequence alignments that are commonly used to infer primary transcript structures from cDNAs or ESTs. One can install and run all algorithms locally, while web-based software tools are also available, for e.g. ASPIC, GENESEQER, MGALIN, and SIM4.

Algorithms for spliced-sequence alignments

ASPIC	http://t.caspur.it/ASPIC/
DDS/GAP2	http://www.tigr.org/software/alignment.shtml
EST_GENOME	http://www.well.ox.ac.uk/~rmott/ESTGENOME/est_genome.shtml
EXALIN	http://blast.wustl.edu/exalin
GENESEQER	http://deepc2.psi.iastate.edu/cgi-bin/gs.cgi
GMAP	http://www.gene.com/share/gmap
MGALIGN	http://origin.bic.nus.edu.sg/mgalign/
MRNAVSGEN	http://genes.mit.edu/genoa
SIM4	http://globin.cse.psu.edu/html/docs/sim4.html
SPA	http://www.biozentrum.unibas.ch/~nimwegen/cgi-bin/spa.cgi
SPIDEY	http://www.ncbi.nlm.nih.gov/spidey
WABA	http://hgwdev-hiram.cse.ucsc.edu/IntronWS120/index.html
