# Supplementary material for: Strategies for Identifying RNA Splicing Regulatory Motifs and Predicting Alternative Splicing Events
Source: PLoS Comput Biol. 2008 Jan 25;4(1):e21. doi: 10.1371/journal.pcbi.0040021 (PMC2217580; doi:10.1371/journal.pcbi.0040021)
Supplement: Table S2 — (42 KB PDF) [file pcbi.0040021.st002.pdf]

Table 2. Selection and overview of several comprehensive motif searches for predicted exonic splicing enhancer (ESE) or silencer (ESS) sequences in mammalian genomes, including ESE-FINDER [40], RESCUE-ESE [28], FAS-ESS [27], PESE/PESS [42], ESR [43], ISRE [44], NI-ESE/NI-ESS elements [46]. All methods are based on the under- or overrepresentation of specific penta- to octamers in exons or introns, and all but one are based on consensus oligonucleotides to search for splicing signals (ESE-FINDER uses position-specific scoring matrices). More differences exist regarding the discrimination of predicted elements (e.g., exon versus intron, exon versus pseudoexon, non-coding versus coding exon) as well as in the extent of phylogenetic conservation.

**Motif search**	**Features**				
	**Background**	**Size**	**Experimentally verified/predicted**	**Species**	**Availability**
ESE-FINDER	SELEX-derived position-specific weight matrices for the family of SR proteins: ASF/SF2, SRp40, SC35, and SRp55	4	4/4	*H.sapiens*	Web server http://rulai.cshl.edu/tools/ESE [40]
RESCUE-ESE/ISE	Hexamer nucleotides statistically enriched in constitutive exons, with weak to moderate splice site strengths, versus introns	238	10/238	*H.sapiens* *M.musculus* *F.rubripes*	Complete set from RESCUE-ESE web server, http://genes.mit.edu/burgelab/rescue-ese [28]
FAS-ESS	GFP-based reporter system for detecting splicing-silencing activity, using a pool of random decamer nucleotides	133	14/21 heterologous exon context	*H.sapiens*	Complete set from FAS-ESE web server, http://genes.mit.edu/fas-ess/ [27]
PESE (PESS)	Octamer nucleotides statistically enriched in internal non-coding exons versus both pseudoexons and exons in 5′ UTRs	2,069 (974)	10/12 (8/8) minigene construct	*H.sapiens*	Complete set from authors' web site [42]
ESR	Dicodon (hexamer) nucleotides statistically enriched and evolutionary conserved in exons of orthologous genes	285	10/285 minigene construct	*H.sapiens* *M.musculus*	Supplementary material [43]
ISRE	Penta-, hexa- and heptamer nucleotides statistically enriched and evolutionary conserved in intronic regions flanking exons of orthologous genes	314	61/68 minigene construct	*H.sapiens* *M musculus* *C familiaris* *R.norvegicus*	Supplementary material [44]
NI-ESE (NI-ESS)	Hexamer nucleotides with ESE or ESS activity, based on the sequence “closeness" of displayed among previously determined RESCUE-ESE and PESE as well as FAS-ESS and PESS sets	*313 (110)<br>* NI cutoff: 0.8 (-0.8)	9/24 minigene construct	*H.sapiens*	Supplementary material [46]
