# Supplementary material for: Strategies for Identifying RNA Splicing Regulatory Motifs and Predicting Alternative Splicing Events
Source: PLoS Comput Biol. 2008 Jan 25;4(1):e21. doi: 10.1371/journal.pcbi.0040021 (PMC2217580; doi:10.1371/journal.pcbi.0040021)
Supplement: Table S3 — (45 KB PDF) [file pcbi.0040021.st003.pdf]

Table 3. All-against-all sequence comparison of different classes of predicted *cis*-elements, described in Table 2. The distance between oligonucleotides (of size k) was measured by the Hamming-like measure h (as applied in Fairbrother *et al*. [28] or Stadler *et al*. [46]); in case of unequal element sizes, the shorter element was compared against all sub-elements in the longer one, and the minimum of h was taken. The average weighted distance (d) was computed as the ratio of the sum over all observed distances times the frequency of occurrence (h x f), over the sum of all frequencies (f), and normalized to the element size and corrected for identical comparisons. Specifically, 1) for two sets (with elements $a_{i=1,2,\ldots,|\ |}$) and /\\ (with $b_{j=1,2,\ldots,|/\backslash|}$), compute the pair-wise distance h_{ij} for any a_i (i = 1,2,…,| |) against all b_j; 2) obtain the weighted-average distance d(,/\\) by computing the sum over the product of all observed values $h_{l=0,1,\ldots,k}$ times their frequencies of occurrence $f(h_l)$, normalized to the sum over all frequencies of occurrence $f(h_l)$; and 3) normalize by the maximum size of compared oligonucleotides, k, and 4) use $d/2$ in case of comparisons between identical sets. For each pair of compared sets, shuffled data were generated by randomly selecting half of each set and subsequent exchange to produce equally mixed sets. A high similarity results in low values (close to d = 0), whereas a high dissimilarity results to high values (close to d = 1). The upper (lower) triangle corresponds to 100 x d for the original (shuffled) data.

	**RESCUE-ESE**	**FAS-HEX2**	**PESE**	**PESS**	**ESR**	**ISRE**	**NI-ESE**	**NI-ESS**
**RESCUE-ESE**	25.5	**64.2**	**50.3**	**57.5**	**58.5**	**59.1**	**55.4**	**65.0**
**FAS-HEX2**	(57.6)	25.6	**57.8**	**53.8**	**61.6**	**57.8**	**64.7**	**55.4**
**PESE**	(70.5)	(72.7)	28.2	**64.5**	**53.2**	**56.6**	**50.6**	**60.2**
**PESS**	(66.4)	(66.5)	(59.7)	26.8	**56.2**	**49.8**	**60.3**	**48.1**
**ESR**	(56.8)	(58.7)	(70.4)	(65.6)	28.8	**58.0**	**58.7**	**61.4**
**ISRE**	(55.5)	(54.5)	(72.1)	(63.5)	(56.7)	24.6	**60.7**	**52.9**
**NI-ESE**	(54.6)	(59.1)	(69.4)	(66.5)	(57.5)	(57.6)	27.5	**65.9**
**NI-ESS**	(57.1)	(53.4)	(73.9)	(67.1)	(58.8)	(51.6)	(59.1)	25.4
