# Supplementary material for: Strategies for Identifying RNA Splicing Regulatory Motifs and Predicting Alternative Splicing Events
Source: PLoS Comput Biol. 2008 Jan 25;4(1):e21. doi: 10.1371/journal.pcbi.0040021 (PMC2217580; doi:10.1371/journal.pcbi.0040021)
Supplement: Table S4 — (59 KB PDF) [file pcbi.0040021.st004.pdf]

Table 4. Selection of actively maintained AS databases. Most databases have been constructed from automated large-scale comparisons of transcript sequences to genomic DNA of known genes, following ENSEMBL, UNIGENE, SWISS-PROT annotation and nomenclature, whereas “hybrid-like” approaches attempt to curate AS data by searches of published research. Databases are purposefully different and serve different users needs: they do so in the scope of interrogated genomes and sequence data, splicing conservation, annotation of functional elements, data download, and several other features (last column). They show further differences in their inference of AS events, for reasons such as different primary data, rules to detect splice patterns, and filter criteria.

**Database**	**http://**	**Features**				
		**Download**	**Genomes**	**Splicing conservation**	**Functional elements**	**Other features**
ASAP II	www.bioinformatics.ucla.edu/ ASAP2	Data and feature tables, in SQL format	Nine vertebrates, including *H.sapiens* and *M.musculus*, four insects, and one nematode	Orthologous exons and introns	-	AS types; EST-derived tissues; cancer tissues; software projected for graphical visualization; search by identifiers, gene name or description; documentation
ASDB	hazelton.lbl.gov/ ~teplitski/alt	-	Many species	-	-	Two divisions: SWISS-PROT protein and GENBANK nucleic acids sequences; search by Medline or database identifers
ASD	www.ebi.ac.uk/ asd	Data tables, in FASTA format	Two mammals: *H.sapiens* *M.musculus*	-	-	Two divisions: ASD (curated) and AST; tools for splice signal analysis
DEDB	proline.bic.nus.edu.sg/ dedb	Splicing graphs, in XML format	*Drosophila melanogaster*	-	-	AS types; search by BLAST or FLYBASE gene name or symbol; documentation
EASDB	eased.bioinf.mdc-berlin.de		Six vertebrates, one insect, nematode and plant	-	-	Tissue, cancer and library classes; search by GENBANK accession number, gene name or description; graphical visizlization; documentation
ECGENE	genome.ewha.ac.kr/ ECgene/gbr					
HOLLWYOOD	hollywood.mit.edu	Data and features from web interface for up to 5,000 exons; complete raw dataset, from FTP server	Two mammals: *H.sapiens* *M.musculus*	Exon-skipping conserved splice variants in orthologous exons	Motif and positional annotation of RESCUE-ESEs and FAS-ESS nucleic acids elements in constitutive and AS exons	AS types; EST-derived tissues; splice-site scores; graphical visualization of representative transcripts; splice site scores; overlay onto UCSC; search by ENSEMBL identifiers, gene symbol or description; documentation
MAASE	maase.genomics.purdue.edu	-	Two mammals: *H.sapiens* *M.musculus*	-	-	Curated annotation of AS events and types; graphical visualization; search by gene name, MAASE or SWISS-PROT identifier registration required;
PASLDB	ymbc.ym.edu.tw/palsdb	-	Two mammals: *H.sapiens* *M.musculus*, and one nematode	-	-	Search by gene name or description; GO terms; graphical visualization; documentation
PROSPLICER	prosplicer.mbc.nctu.edu.tw	-	*H.sapiens*	-	-	Search by gene name, ENSEMBL, SWISS-PROT or other identifiers; documentation
SPLICEINFO	spliceinfo.mbc.nctu.edu.tw	Data pertinent to *H.sapiens*			Tool for motif search and/or analysis (SpliceMotif)	Search by gene name, ENSEMBL, SWISS-PROT or other identifiers; Tissue categories; documentation
SPLICENEST	splicenest.molgen.mpg.de	-	Two mammals: *H.sapiens* *M.musculus*, one fly and plant	-	-	Web-based display and exploration tool for AS; search by UNIGENE or clone identifier, or gene accession number; documentation
STACKDB	ww2.sanbi.ac.za/ Dbases.html	Database download	*H.sapiens*	-	-	Graphical visualization; multiple disease and one tissue category; registration required
FASTDB	www.fast-db.com/ fastdb2/frame.html	-	Two mammals: *H.sapiens* *M.musculus*	-	Tools for related bioinformatics searches	Graphical visualization; search by various names and identifiers; batch upload; documentation

Dirk **Holste**, Uwe **Ohler**
