# Supplementary material for: Strategies for Identifying RNA Splicing Regulatory Motifs and Predicting Alternative Splicing Events
Source: PLoS Comput Biol. 2008 Jan 25;4(1):e21. doi: 10.1371/journal.pcbi.0040021 (PMC2217580; doi:10.1371/journal.pcbi.0040021)
Supplement: Table S5 — (32 KB PDF) [file pcbi.0040021.st005.pdf]

Table 5. Selection of several algorithms for *ab initio* detection of alternative exons and/or intron-retention events. The table contains examples for both exon classification and exon discovery.

**Algorithms for predicting alternative exons**	**Contact**
Prediction of ~1,000 AS events involving known exons that are conserved between *H.sapiens* and *M.musculus*, based on feature selection and support vector machine classification [57]	**E-mail**: gideon@mta.ac.il
Classification of orthologous and splicing-conserved alternative exons in *Drosophila melanogaster* and *D.pseudoobscura*, which predicted ~160 alternative exons (various AS types) in the fly [55]	**Website**: http://genetics.uchc.edu/Graveley
**ACESCAN**: Classification of orthologous and alternative splicing-conserved exons (ACEs); predicted ~2,000 skipped ACEs in *H.sapiens* and *M.musculus* genes, based on motifs and generic sequence features, with experimentally validated specificity of ~70%. [50]	**Webserver**: http://genes.mit.edu/acescan
**UNCOVER**: PairHMM to discover new AS events (skipped exons and retention-type introns) that are conserved in *H.sapiens* and *M.musculus,* which predicted ~50 novel skipped exons in the ENCODE region and genome-wide about two dozen retained introns [59]	**Software**: http://genes.mit.edu/burgelab/software.html
**RASE**: Application of support vector machines to classify or discover skipped exons in *Caenorhabditis.elegans*, which predicted ~200 candidate alternative exons. [54]	**Software**: http://www.fml.tuebingen.mpg.de/raetsch/projects/RASE
**PASE**: Discovery of skipped exons including signal peptide sequences, which validated of 33 new *C.elegans* and 26 new *H.sapiens* alternative exons [64]	**E-mail**: rmitra@genetics.wustl.edu
Prediction of new mRNA isoforms involving AS events of both exon skipping and retention-type introns, based on the presence of PFAM protein domains [63]	**E-mail**: hiller@informatik.uni-freiburg.de
